# Effects of atropine and tropicamide on ocular biological parameters in children: a prospective observational study

**DOI:** 10.1186/s12886-023-02840-5

**Published:** 2023-03-13

**Authors:** Yulin Tao, Mohan Li, Jian Tan, Jing Huang, Xiaokang Cheng, Ping Xie, Xiansheng Liu, Qiong Zhou, Jun Ouyang

**Affiliations:** 1 Department of Ophthalmology, Jiujiang No 1 Peoples Hospital, Affiliated Jiujiang Hospital of Nanchang University, 48 South Taling Road, Jiujiang, 332000 Jiangxi China; 2 Department of Ophthalmology, The First Affiliated Hospital of Nanchang University, Jiangxi Center of National Ocular Disease Clinical Research Center, 17 Yongwai Main Street, Nanchang, 330006 Jiangxi China; 3 Department of Ophthalmology, The Second Hospital of Anhui Medical University, No.678 Furong Road, Economic Development Zone, Hefei, 230031 Anhui China

**Keywords:** Ocular biometry, Cycloplegia, Atropine, Tropicamide, Children

## Abstract

**Background:**

The effectiveness of cycloplegia in delaying the progression of myopia and its application in refractive examination in children have been extensively studied, but there are still few studies on the effects of atropine/tropicamide on ocular biological parameters. Therefore, the purpose of this study was to explore the effects of atropine/tropicamide on children's ocular biological parameters in different age groups and the differences between them.

**Methods:**

This was a prospective observational study in which all school children were examined for dioptres and ocular biological parameters in the outpatient clinic, and 1% atropine or tropicamide was used for treatment. After examination, we enrolled the patients grouped by age (age from 2 to 12 years treated by atropine, 55 cases; age from 2 to 10 years treated by tropicamide, 70 cases; age from 14 to 17 years treated by tropicamide, 70 cases). The ocular biological parameters of each patient before and after cycloplegia were measured, and the difference and its absolute value were calculated for statistical analysis using an independent-samples t test.

**Results:**

We compared the value and the absolute value of the differences in ocular biological parameters before and after cycloplegia in the same age group, and we found that the differences were not statistically significant (*P* > 0.05). There were significant differences in the corresponding values of AL, K1 and ACD among the different age groups (*P* < 0.05). Before cycloplegia, there were significant differences in AL, K, K1, K2 and ACD in different age groups (*P* < 0.05). However, the differences in AL, K, K1, K2 and ACD among different age groups disappeared after cycloplegia (*P* > 0.05).

**Conclusions:**

This study demonstrated that atropine/tropicamide have different effects on cycloplegia in children of different ages. The effects of atropine/tropicamide on ocular biological parameters should be fully considered when evaluating the refractive state before refractive surgery or mydriasis optometry for children of different ages.

## Highlights

1. Cycloplegic drugs have effects on the ocular biological parameters of children at different ages.

2. The effects of different types of cycloplegic drugs were almost the same in children within the same age groups.

3. There were significant differences in the effects of different types of cycloplegic drugs on ocular biological parameters in children of different age groups.

## Introduction

Myopia is caused by a late elongation of the eyeball, which can cause light the focus only in front of the retina, not on it. The incidence of myopia in East Asian countries has risen rapidly in the past 50 years [1]. Myopia seriously threatens people's physical and mental health and even leads to blindness, especially in cases of high myopia. The prevalence of myopia worldwide is almost 2 billion people, accounting for approximately 28.3% of myopia cases [2]. Simultaneously, approximately 277 million people have high myopia, approximately 4.0% of the worldwide population [2]. The incidence of myopia and high myopia worldwide is also increasing yearly [2, 3]. Refraction error is the primary cause of vision loss in children and adolescents, and the prevention and treatment of myopia are facing serious challenges in China [4–6]. On all accounts, the accurate diagnosis and appropriate treatment of myopia is increasingly important.

Mydriasis optometry with ciliary paralysis is the most commonly used method to detect refractive dioptres in children [7–9]. The ciliary paralysis reagents mainly used in China are atropine as a long-acting agent and tropicamide as a short-acting agent. However, previous studies have shown that cycloplegia has a certain impact on ocular biological parameters, especially in children, including refractive state [10–13], axial length (AL) [11, 13–16], corneal curvature, axial position of corneal astigmatism, central corneal thickness (CCT) [10, 15–19], anterior chamber depth (ACD) [11, 13, 15–17, 20–25], anterior chamber volume (ACV) [17, 22], and white-to-white (WTW) distance [16, 23] of the participants. These effects will change the refractive state and ocular biological parameters of patients, which will further affect the accuracy of preoperative measurement of corneal refractive surgery. With the increasing number of myopic patients and the stringent requirements for visual quality after surgery, an increasing number of children need to undergo orthokeratology, which highlights the importance of the accurate evaluation of preoperative optometry [26–31]. The main purpose of this study was to explore the differences in the effects of the two drugs on biological parameters to provide a more reliable reference for the results of optometry before keratoplasty or atropine application. The application of low-concentration atropine worldwide to control myopia in children and the exploration of the best concentration make the study of the influence of drugs on biological parameters more important.

Atropine and tropicamide have different effects on ocular biological parameters due to their different pharmacological characteristics. At present, there is no research on which cycloplegic can be used for optometry at different ages to minimize the impact on ocular biological parameters and no research on the differences in the effects on ocular biological parameters between the two drugs. In this study, a prospective observation method was used to compare and draw relevant conclusions among patients of the same age groups and different age groups who used atropine or tropicamide. Ocular biological parameters were obtained before and after the application of atropine/tropicamide in these school children.

## Methods

### Study participants

We enrolled 195 patients with refractive error in this prospective observational study, and only the right eye of each patient was included. The implementation of this study followed the Declaration of Helsinki. This study was also approved by the ethics committee of the First People's Hospital of Jiujiang City in China. Furthermore, written informed consent was obtained from each participant and their legal guardian at the time of study enrolment. We divided all patients into three groups. The first group was treated by atropine and included 55 children aged 2–12 years (mean age, 5.38 ± 2.44 years), the second group was treated by tropicamide and included 70 children aged 2–10 years (mean age, 5.59 ± 2.45 years), and the third group was also treated by tropicamide and included 70 children aged 14–17 years (mean age, 15.27 ± 1.01 years). We compared the changes in the ophthalmic biological parameters before and after cycloplegia and evaluated the effects of the application of the two types of ciliary muscle paralysis agents. All children were recruited from the outpatient clinic, including 91 males and 104 females. Those who conformed to the following criteria were included in this study: age-appropriate patients with no eye diseases found after systematic eye examination (vision, FSLB, fundus examination, etc.). The exclusion criteria were as follows: suffering or having a history of eye diseases; suffering from chronic diseases such as asthma and heart disease; and wearing corneal contact lenses in the past month. Systematic ophthalmological examination was administered to the children, including ocular anterior segment examination, eye vision, best-corrected visual acuity (BCVA), functional slit lamp biomicroscopy (FSLB), intraocular pressure (IOP) and fundus examination, which were performed by an ophthalmologist to rule out organic ophthalmopathy.

### Ocular examinations

Ocular examination was performed in the hospital after patients were screened for abnormal vision when they were at school. Ocular biological parameters were measured before and after cycloplegia with a noncontact apparatus (AL-Scan, NIDEK CO., LTD., Aichi Prefecture, Japan). We asked the patients to stare at the detection beam during the measurement. Both eyes were measured each time, but only the right eye was included because of the high correlation between right and left eyes of the same individual [4]. We measured the mean keratometry (K), flat keratometry (K1), steep keratometry (K2), axis, CCT, ACD and WTW of each patient only once, whereas 5 measurements of AL were generated automatically and averaged. The system software automatically calculated the values of CCT and ACD through image analysis. ACD was defined as the distance from the anterior corneal pole to the anterior lens surface. Computer optometry was performed on the patients before and after cycloplegia using a desktop autorefractor (RM8900; Topcon Corp., Tokyo, Japan); measurements were repeated 3 times in each eye, and the average value was recorded. All data were artificially extracted from the device and filled in a spreadsheet file. We assigned a unique identification number for each patient throughout the study.

Fifty-five children aged 2 to 12 years old were administered 1% atropine sulfate eye gel to induce cycloplegia 3 times a day, and their eyes were treated for 3 consecutive days. After 3 days, they were revisited, and optometry was performed. Seventy age-matched children from 2 to 10 years were treated with tropicamide phenylephrine eye drops (Mydrin-P). Another 70 age-unmatched children from 14 to 17 years were also treated with Mydrin-P. The tropicamide drop was administered 4 times in one day every 5 min. We evaluated cycloplegia and pupil dilation in the patients after an additional 20 min. Cycloplegia was considered complete when the pupil was dilated to at least 6 mm and a pupillary light reflex was absent [4, 32]. Ultimately, we evaluated the indicators AL, K, K1, K2, axis, CCT, ACD and WTW.

### Statistical analysis

We compared the atropine group with the age-matched tropicamide group and the atropine group with the age-unmatched tropicamide group. All continuous data we gained from the research were expressed as the mean ± standard deviation (x̅ ± s). The Kolmogorov‒Smirnov test was performed to detect the normality of the data. One-way ANOVA was used for the homogeneity test of variances. The independent-samples t test was used for statistical analysis between the independent groups when the continuous data were normally distributed. The constituent ratios of counting data (for example, gender) were analysed by χ^2^ test. Two-tailed *P* values were used in all analyses. The 95% confidence interval (95% CI) of the difference was calculated for the comparison of different independent samples. A *p* value of < 0.05 was considered statistically significant. All statistical analyses were performed in SPSS 22.0 software (IBM Corp., Armonk, New York, USA).

## Results

In this study, we compared the effects of atropine and tropicamide on ocular biological parameters in the same age groups, which were defined as under 13 years old treated with atropine and under 13 years old treated with tropicamide (*P* > 0.05 for age statistical analysis), and in the different age groups, which were defined as under 13 years old treated with atropine and over 13 years old treated with tropicamide (*P* < 0.05 for age statistical analysis) (Table 1). Fifty-five patients were enrolled in the atropine group, and 70 patients were enrolled in each of the tropicamide groups. There was no significant difference in sex among the three groups (*P* > 0.05) (Table 1). We calculated the differences in ocular biological parameters after ciliary paralysis, that is, the value after cycloplegia minus the value before cycloplegia, and recorded the absolute value of the difference between the two measurements. The difference reflects the overall trend of these parameters, and increases and decreases may cancel each other out. The absolute value reflects the absolute trend, and increases or decreases in parameters were regarded as the same trend. The differences and absolute values of ocular biological parameters were all compared in the same age groups and different age groups. In this study, the biometric parameters of the eyes of patients were as follows: AL, K, K1, K2, axis, CCT, ACD and WTW.

**Table 1:** The distribution of cases, gender, and age of the three different groups

	Atropine	Tropicamide	95% CI	*P* value
Cases (N)	55	70 (70)		
Gender (n)	24 (31)	35 (35)		0.479^#^
		32 (38)		0.817^#^
	Mean ± SD			
Age (y)	5.38 ± 2.44	5.59 ± 2.45	-1.08 to 0.67	0.644^&^
		15.27 ± 1.01	-10.53 to -9.25	< 0.001^&^*

*N/n* Number, *y* Year, *95% CI* 95% confidence interval of the difference, *Mean* Mean value, *SD* Standard deviation

^#^χ^2^ test, ^&^Independent-samples t test, *P < 0.05 indicates statistical significance

We found that there were no significant differences in the value of each parameter before and after atropine/tropicamide treatment within the same age group (*P* > 0.05) (Table 2). However, the differences in AL and ACD after the application of atropine/tropicamide in different age groups were statistically significant (*P* < 0.05) (Table 2). AL and ACD increased in patients under 13 years of age treated with atropine (mean ± standard deviation, 1.71 ± 2.38 and 0.42 ± 0.42, respectively), and they decreased in patients over 13 years of age treated with tropicamide (mean ± SD, -1.47 ± 2.13 and -0.01 ± 0.34, respectively) (Table 2).

**Table 2:** Comparison of the differences in ocular biological parameters before and after atropine/tropicamide in the same age groups and different age groups

Parameter	Atropine (M ± SD)	Tropicamide		95% CI	*P* value
		Age Range (y)	M ± SD		
AL (mm)	1.71 ± 2.38	2–10	1.37 ± 2.32	-0.50 to 1.17	0.427
		14–17	-1.47 ± 2.13	2.38 to 3.98	< 0.001*
K (D)	0.39 ± 2.54	2–10	0.31 ± 2.36	-0.79 to 0.95	0.859
		14–17	-0.07 ± 1.98	-0.33 to 1.27	0.252
K1 (D)	0.29 ± 2.52	2–10	0.43 ± 2.30	-1.00 to 0.71	0.746
		14–17	-0.20 ± 1.91	-0.29 to 1.27	0.216
K2 (D)	0.49 ± 2.76	2–10	0.23 ± 2.62	-0.69 to 1.22	0.581
		14–17	0.06 ± 2.33	-0.46 to 1.34	0.339
Axis (d)	21.95 ± 128.54	2–10	13.96 ± 111.80	-34.61 to 50.59	0.711
		14–17	-18.04 ± 121.83	-4.53 to 84.51	0.078
CCT (μm)	9.29 ± 41.02	2–10	8.93 ± 47.80	-15.97 to 16.69	0.965
		14–17	7.29 ± 46.12	-14.00 to 18.00	0.805
ACD (mm)	0.42 ± 0.42	2–10	0.36 ± 0.37	-0.14 to 0.25	0.561
		14–17	-0.01 ± 0.34	0.24 to 0.61	< 0.001*
WTW (mm)	0.08 ± 0.60	2–10	0.13 ± 0.63	-0.26 to 0.18	0.725
		14–17	0.08 ± 0.49	-0.19 to 0.20	0.947

The independent-samples t test was used in this table

*y* Year, *mm* Millimetre, *D* Dioptre, *d* Degree, *μm* Micron, *95% CI* 95% Confidence interval of the difference, *M* Mean value, *SD* Standard deviation

^*^P < 0.05 indicates statistical significance

We found the same trend in the absolute difference value of each parameter in the different age groups compared to the same age groups post-cycloplegia (*P* > 0.05) (Table 3). The absolute values of K1 and ACD were statistically significant after cycloplegia in the two groups (*P* < 0.05) (Table 3). The study showed that K1 and ACD increased after treatment with atropine/tropicamide in the different age groups (Table 3).

**Table 3:** Comparison of the absolute value of the difference in ocular biological parameters before and after atropine/tropicamide in the same age groups and different age groups

Parameter	Atropine (M ± SD)	Tropicamide		95% CI	*P* value
		Age Range (y)	M ± SD		
AL (mm)	2.26 ± 1.86	2–10	2.20 ± 1.54	-0.55 to 0.66	0.857
		14–17	1.98 ± 1.65	-0.35 to 0.90	0.389
K (D)	2.04 ± 1.54	2–10	1.91 ± 1.41	-0.40 to 0.65	0.636
		14–17	1.59 ± 1.17	-0.04 to 0.93	0.069
K1 (D)	2.03 ± 1.49	2–10	1.87 ± 1.39	-0.36 to 0.67	0.550
		14–17	1.52 ± 1.15	0.04 to 0.98	0.034*
K2 (D)	2.24 ± 1.67	2–10	2.06 ± 1.61	-0.40 to 0.76	0.543
		14–17	1.86 ± 1.39	-0.16 to 0.92	0.168
Axis (d)	101.73 ± 80.45	2–10	79.16 ± 79.62	-5.96 to 51.10	0.120
		14–17	92.61 ± 80.44	-19.58 to 37.81	0.531
CCT (μm)	30.33 ± 28.86	2–10	37.87 ± 30.18	-18.28 to 3.19	0.167
		14–17	37.67 ± 27.22	-17.50 to 2.82	0.155
ACD (mm)	0.48 ± 0.34	2–10	0.44 ± 0.28	-0.10 to 0.19	0.554
		14–17	0.27 ± 0.20	0.09 to 0.34	0.001*
WTW (mm)	0.45 ± 0.39	2–10	0.50 ± 0.40	-0.19 to 0.10	0.525
		14–17	0.45 ± 0.39	-0.07 to 0.18	0.384

The independent-samples t test was used in this table

*y* Year, *mm* Millimetre, *D* Dioptre, *d* Degree, *μm* Micron, *95% CI* 95% confidence interval of the difference, *M* Mean value, *SD* Standard deviation

^*^P < 0.05 indicates statistical significance

There were no significant differences in the ophthalmic biological parameters before atropine/tropicamide treatment in the same age groups (*P* > 0.05) (Table 4). The parameters AL, K, K1, K2 and ACD in the different age groups were statistically significant before atropine/tropicamide (*P* < 0.05) (Table 4). When these children were treated with atropine/tropicamide, there were no significant differences in any of the ocular biological parameters, whether in the same age groups or in different age groups (*P* > 0.05) (Table 5).

**Table 4:** Comparison of the ocular biological parameters before atropine/tropicamide in the same age groups and different age groups

Parameter	Atropine (M ± SD)	Tropicamide (M ± SD)	95% CI	*P* value
AL (mm)	21.60 ± 1.37	22.02 ± 1.35	-0.91 to 0.06	0.085
		24.79 ± 1.49	-3.71 to -2.68	< 0.001*
K (D)	42.71 ± 1.83	42.90 ± 1.57	-0.79 to 0.42	0.540
		43.47 ± 1.44	-1.34 to -0.18	0.010*
K1 (D)	41.85 ± 1.79	41.97 ± 1.59	-0.73 to 0.47	0.673
		42.66 ± 1.47	-1.39 to -0.24	0.006*
K2 (D)	43.58 ± 1.97	43.79 ± 1.73	-0.87 to 0.44	0.512
		44.29 ± 1.60	-1.34 to -0.08	0.028*
Axis (d)	93.55 ± 83.23	94.71 ± 82.47	-30.70 to 28.37	0.938
		106.01 ± 82.42	-41.99 to 17.05	0.405
CCT (μm)	550.06 ± 27.09	548.71 ± 32.73	-9.70 to 12.38	0.810
		551.14 ± 33.79	-12.39 to 10.22	0.849
ACD (mm)	3.37 ± 0.36	3.39 ± 0.26	-0.16 to 0.12	0.824
		3.81 ± 0.21	-0.57 to -0.31	< 0.001*
WTW (mm)	11.75 ± 0.53	11.70 ± 0.39	-0.12 to 0.21	0.589
		11.75 ± 0.53	-0.17 to 0.15	0.888

The independent-samples t test was used in this table

*mm* Millimetre, *D* Dioptre, *d* Degree, *μm* Micron, *95% CI* 95% confidence interval of the difference, *M* Mean value, *SD* Standard deviation

^*^P < 0.05 indicates statistical significance

**Table 5:** Comparison of the ocular biological parameters after atropine/tropicamide in the same age groups and different age groups

Parameter	Atropine (M ± SD)	Tropicamide (M ± SD)	95% CI	*P* value
AL (mm)	23.31 ± 1.84	23.40 ± 1.65	-0.71 to 0.53	0.780
		23.32 ± 1.58	-0.62 to 0.59	0.970
K (D)	43.10 ± 1.40	43.21 ± 1.55	-0.64 to 0.42	0.686
		43.40 ± 1.29	-0.77 to 0.18	0.221
K1 (D)	42.14 ± 1.42	42.41 ± 1.55	-0.80 to 0.26	0.315
		42.46 ± 1.24	-0.79 to 0.15	0.180
K2 (D)	44.07 ± 1.55	44.02 ± 1.67	-0.53 to 0.63	0.864
		44.34 ± 1.55	-0.83 to 0.28	0.333
Axis (d)	115.49 ± 80.44	108.67 ± 82.03	-22.19 to 35.83	0.643
		87.97 ± 83.73	-1.84 to 56.87	0.066
CCT (μm)	559.35 ± 29.18	557.64 ± 35.98	-10.35 to 13.76	0.780
		558.43 ± 29.30	-9.72 to 11.55	0.866
ACD (mm)	3.39 ± 0.25	3.75 ± 0.29	-0.10 to 0.18	0.560
		3.80 ± 0.27	-0.15 to 0.12	0.818
WTW (mm)	11.83 ± 0.38	11.83 ± 0.51	-0.16 to 0.17	0.937
		11.84 ± 0.36	-0.14 to 0.13	0.964

The independent-samples t test was used in this table

*mm* Millimetre, *D* Dioptre, *d* Degree, *μm* Micron, *95% CI* 95% confidence interval of the difference, *M* Mean value, *SD* Standard deviation

^*^P < 0.05 indicates statistical significance

## Discussion

Ciliary paralysis agents are increasingly widely used in ophthalmology. They were mainly used for dioptre examination in the past, but currently, they can be used to delay the development of myopia in children [33–35]. There are three main kinds of ciliary muscle paralysis agents used in the clinic: atropine, tropicamide and cyclopentolate [8]. Current research is mainly focused on the control of myopia in children with low concentrations of atropine [36]. Wei S et al. [37] conducted a study on the effect of low-concentration atropine in delaying myopia and eye axis prolongation in children. They enrolled 220 children aged 6 to 12 years with myopia of − 1.00 D to − 6.00 D in both eyes; designed a randomized, placebo-controlled, double-masked study; measured the parameters at baseline, 6 months, and 12 months; and grouped patients in a 1:1 ratio to 0.01% atropine or placebo. Wei S et al. [37] found that the difference in myopia progression was statistically significant at 0.26 D (95% CI, 0.12–0.41; *P* < 0.001) over 1 year, with mean myopia progression of − 0.49 (0.42) D and − 0.76 (0.50) D in the 0.01% atropine and placebo groups, respectively. They also found a relative decrease of 34.2% in myopia headway, and the axial elongation was reduced by 22.0%. However, these changes over 1 year were slight and were limited by approximately 70% follow-up. Saxena R et al. [35] also carried out a multicentric, double-blinded, placebo-controlled randomized clinical trial, followed up for one year, and reported that myopia progression emerged with the application of 0.01% atropine drops in Indian children, but the axial length elongation had no significant difference. Wei S et al. [37] reported no serious adverse events related to atropine among the children. The above studies show that the effect of cycloplegic agents, especially atropine, on myopia is clear, but the difference in the effects of atropine and tropicamide on ocular biological parameters is still unclear. A recent study by Li FF [38] detected the effects of different concentrations of atropine (0.05%, 0.025% and 0.01%) on ocular biological parameters when controlling the progression of myopia compared to placebo over one year. They measured the ocular biometric parameters of cycloplegic spherical equivalent refraction (SER), AL, corneal curvature, and ACD by IOL Master; calculated corneal astigmatism and lens power; and evaluated their associations with the changes in SER. Ultimately, they found that the changes in AL were statistically significant with a concentration-dependent response over one year (*p* < 0.001), the corneal power remained stable (*p* = 0.10), and lens power and ACD were similar across all concentrations (*p* = 0.24 and 0.41, respectively) [38]. They concluded that different low concentrations of atropine had no significant influence on corneal power and lens power in clinical practice, and its main efficacy in reducing the risk of myopia complications was to reduce AL elongation [38].

Through the above studies, we know that the continuous application of low concentrations of atropine will delay the prolongation of AL and control myopia. In this study, we examined the effects of high concentrations of atropine (1%) and tropicamide on ocular biological parameters in children and calculated the changes in parameters before and after cycloplegia (difference: postcycloplegia minus precycloplegia; absolute value of difference: |postcycloplegia minus precycloplegia|). In previous studies, some scholars had confirmed that cycloplegia in children could lead to changes in AL, K, CCT and ACD, among which studies on ACD increased observably [7, 11, 13–16, 18–25]. Similar conclusions were obtained in our study; the ocular biological parameters changed (increased or decreased) to varying degrees after cycloplegia. Most of the previous studies were in response to changes in AL, CCT and ACD. Our study showed that the differences in AL and ACD were significantly different between the atropine group aged 2–12 years and the tropicamide group aged 14–17 years (*P* < 0.05). The absolute value of the difference between the two groups was significant in K1 and ACD (*P* < 0.05). However, in the comparison of the same age groups, there was no statistically significant difference in the changes caused by atropine or tropicamide. The situation changed when we switched our attention to a separate comparison of the parameters before and after cycloplegia. Before cycloplegia, there were no significant differences in ocular biological parameters in the same age groups (*P* > 0.05), but there were significant differences in AL, K, K1, K2 and ACD between different age groups (*P* < 0.05). After cycloplegia, the differences in AL, K, K1, K2 and ACD among different age groups disappeared (*P* > 0.05). This is due to the different intensity and duration of atropine and tropicamide on the ciliary nerve, and there are significant differences in the response of children of different ages to these two kinds of ciliary paralysis agents. Therefore, the difference before cycloplegia was offset by the application of cycloplegia.

In this study, we included a certain number of patients and divided them into different age groups. We have obtained some scientific results that are different from those of our predecessors. The conclusions we obtained have definite reference value. A limitation of our study was that a group using atropine in older children was not included because atropine is rarely used for mydriasis in these individuals, which remains to be further discussed.

In conclusion, this study demonstrated that atropine and tropicamide have different effects on cycloplegia in children of different ages. Atropine is recommended for mydriasis optometry in young children due to its stronger effect on ciliary paralysis. The effects of these two drugs on children's ocular biological parameters are also different, and both will lead to different changes in the parameters. The effects of atropine/tropicamide on ocular biological parameters should be fully considered when evaluating the refractive state before refractive surgery or calculating the degree of intraocular lens before cataract surgery. In view of the significance of this study, we hope to include more patients of other ages for further study in the future.

## Data Availability

All data generated or analysed during this study are included in this published article. All data are available upon request; please contact Yulin Tao and Jun Ouyang to request the data.
